# Synthesis and Formation Mechanism of Limestone-Derived Porous Rod Hierarchical Ca-based Metal–Organic Framework for Efficient CO_2_ Capture

**DOI:** 10.3390/ma13194297

**Published:** 2020-09-26

**Authors:** Po-Hsueh Chang, Hua-Pei Hsu, Szu-Chen Wu, Cheng-Hsiung Peng

**Affiliations:** 1 Department of Chemical and Materials Engineering, Minghsin University of Science and Technology, Xinxing Road, Xinfeng, Hsinchu 30401, Taiwan; pohsueh.chang@gmail.com; 2 Department of Materials Science and Engineering, National Chiao Tung University, 1001 University Road, Hsinchu 30010, Taiwan; ivy810823@gmail.com (H.-P.H.); s320431@hotmail.com (S.-C.W.)

**Keywords:** limestone, metal–organic framework, CO_2_ capture, carbonation–calcination cycles, oriented attachment growth

## Abstract

Limestone is a relatively abundant and low-cost material used for producing calcium oxide as a CO_2_ adsorbent. However, the CO_2_ capture capacity of limestone decreases rapidly after multiple carbonation/calcination cycles. To improve the CO_2_ capture performance, we developed a process using limestone to transform the material into a rod Ca-based metal–organic framework (Ca-MOF) via a hydrothermal process with the assistance of acetic acid and terephthalic acid (H_2_BDC). The structural formation of rod Ca-MOF may result from the (200) face-oriented attachment growth of Ca-MOF sheets. Upon heat treatment, a highly stable porous rod network with a calcined Ca-MOF-O structure was generated with a pore distribution of 50–100 nm, which allowed the rapid diffusion of CO_2_ into the interior of the sorbent and enhanced the CO_2_ capture capacity with high multiple carbonation–calcination cycle stability compared to limestone alone at the intermediate temperature of 450 °C. The CO_2_ capture capacity of the calcined porous Ca-MOF-O network reached 52 wt% with a CO_2_ capture stability of 80% after 10 cycles. The above results demonstrated that rod Ca-MOF can be synthesized from a limestone precursor to form a porous network structure as a CO_2_ capture sorbent to improve CO_2_ capture performance at an intermediate temperature, thus suggesting its potential in environmental applications.

## 1. Introduction

In the past decade, CO_2_ emissions have been the most important cause of global warming. Calcium-based adsorbents have a high adsorption capacity, and are low cost and non-hazardous, making them candidates for high-temperature solid adsorbents with a carbonation temperature for CaO-based adsorbents at 600–700 °C. However, there are serious problems for calcium-based sorbents because the CO_2_ adsorption capacity decreases rapidly after multiple carbonation–calcination cycles, which can be attributed to particle aggregation and sintering during the high-temperature heating process [1]. There have been several methods proposed to overcome this problem, such as adding inert material [2,3,4], generating pores in the particles [5], decreasing the particle size [6,7,8,9] and enhancing the surface area [10,11]. On the other hand, although these fine particles can provide more surface reaction area for CO_2_ adsorption and desorption, aggregation has been an important challenge. Therefore, their applicability in actual dynamic systems, even at the lab scale, such as fixed/fluidized bed reactors, is very challenging.

Recently, to reduce the loss in CO_2_ capture and increase the capture performance during multiple cycles, porous Ca-based sorbents were developed and modified to enhance the CO_2_ capture capacity and long-term cyclic stability. Wang et al. [12] synthesized a porous spherical adsorbent via a fast precipitation method with the presence of sodium polystyrene sulfonate (PSS). The CO_2_ capture performance can be enhanced by forming novel mesoscopic hollow spheres of CaO/Ca_12_Al_14_O_33_ with a tunable cavity size [13]. It is believed that the void space in the hollow structures can buffer against the local large volume change during carbonation/calcination cycles, and enable the alleviation of the pulverization and aggregation problem of the sorbent material, hence improving the cycling performance. Boyjoo et al. [14] developed special CaCO_3_@C yolk–shell particles that have a basic calcium-based core for the affinity of CO_2_ and a microporous carbon shell for the preferential passage of small molecules with respect to larger sized molecules. Li et al. [15] prepared CaCO_3_@mesoporous silica in a core–shell structure (denoted CaCO_3_@mSiO_2_) as a high-performance CO_2_ sorbent. The improved carbonation conversion retention of the (CaCO_3_@5.6 wt % mSiO_2_)-based pellet sorbent was around 25% after 50 cycles of decarbonation/carbonation, which was higher than that of the CaCO_3_-based sorbent (13%). Although these fine particles can provide more surface reaction area for CO_2_ adsorption and desorption, aggregation has been an important issue. When applying the fine particles in a real calcium looping process, this may become a quite challenging when fluidized bed reactors are typically used. Valverde et al. developed silica agglomerates to act as dispersants of fine Ca (OH)_2_ particles to improve the contact efficiency between the CO_2_ adsorbent and CO_2_ in the fluidized bed [16]. In addition, nanosilica was also used as an additive to increase the gas–solids contact efficiency and, therefore, CO_2_ chemisorption on Ca(OH)_2_ particles can be enhanced in a dry gas–solid carbonation in fluidized beds [17].

**Scheme 1:**
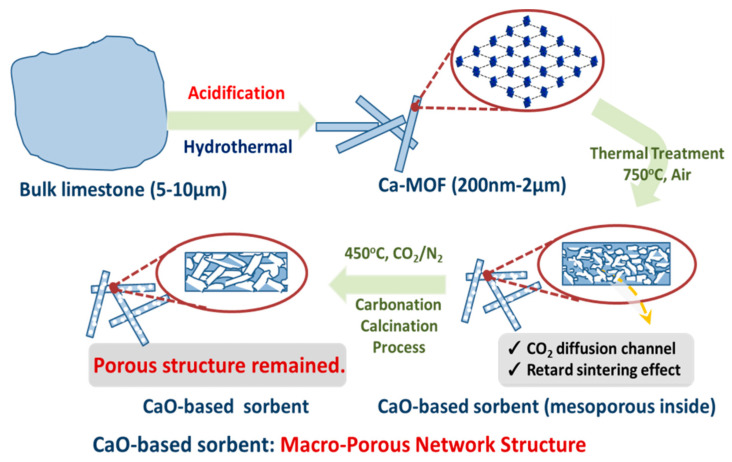
Limestone precursor transformed into porous rod calcium-based sorbents by hydrothermal method and acidification route.

Limestone is a natural mineral, but its CO_2_ capture capacity decreases rapidly after multiple carbonation/calcination cycles. [18] Ridha et al. [19] reported that limestone treated with an organic acid could enhance CO_2_ capture, but still showed cyclic degradation due to the aggregation and partial sintering of CaCO_3_ particles. Recently, Raganati et al. investigated the possibility of enhancing the carbonation performances of fine limestone (<50 μm) or activating carbon in temperature swing, sound-assisted, fluidized-bed reactors to overcome the strong interparticle forces [20,21]. On the other hand, metal–organic frameworks (MOFs) are members of a new class of hybrid organic–inorganic materials that have regular pores ranging from micro- to mesopores, resulting in a large pore surface area, a highly designable framework, and surface functionality [22]. However, the interaction between adsorbed CO_2_ and MOF adsorbents is weak, so the MOF species was considered as a low-temperature CO_2_ adsorbent. On the other hand, as mentioned above, calcium-based adsorbents were usually used as high-temperature solid sorbents at 600–700 °C. Therefore, in this work, we propose a new process to synthesize rod-like Ca-MOF using a limestone precursor by the hydrothermal method and an acidification route to control the nucleation and growth of Ca-MOF using various concentrations of acetic acid. After thermal treatments, rod-like calcium-based sorbents with a porous network structure were formed (Scheme 1), which can significantly improve the CO_2_ capture capacity and long-term cycle stability at an intermediate temperature such as 450 °C because the porous network can form connected multi-channels for gas/solid contact and mass/heat transfer. In addition, the microporous rod CO_2_ adsorbents can avoid close contact like spherical nanoparticles to overcome interparticle forces. To the best of our knowledge, this is the first investigation of a porous rod-like network structure as an intermediate-temperature CO_2_ sorbent achieved by modifying natural limestone. We also provide an assessment of the CO_2_ carbonation/calcination cycle performance of the porous CaO-based rod.

## 2. Experimental Section

### 2.1. Hydrothermal Synthesis of Rod Ca–Metal–Organic Framework

The rod Ca-MOFs were synthesized from limestone (CaCO_3_, Ilan, Taiwan) by a hydrothermal process via the assistance of acetic acid. The morphology and composition of nature limestone are illustrated in Figure S1. First, the ligand solution of 6 mmole terephthalic acid (H_2_BDC, 98%, Alfa Aesar (Ward Hill, MA, USA)) was dissolved in 20 mL of pre-heated N,N-dimethylacetamide (C_4_H_9_NO, DMA, 99.5%, J.T.Baker (Delaware, PA, USA)) at 90 °C until the powders were completely dissolved in 30 min. The metal solution of 6 mmole limestone was then acidified in various concentrations of acetic acid including 1, 5 and 10 M in DI water for 24 h. Next, the ligand solution was added into the metal solution and stirred for 10 min. Finally, the mixture solution was poured into a Teflon-lined stainless-steel autoclave and reacted at 120 °C from 1 to 18 h. In order to collect the powders and remove unreacted ions, the reacted solution was washed with ethanol through high-speed centrifugation 4 times and dried at 60 °C in an oven for 12 h. All the samples were ground to fine powders. The as-synthesized Ca-MOFs were denoted as Ca-MOF-xh-y, where x and y indicated the acidification time of limestone and the concentration of acetic acid, respectively. Subsequently, the raw powders were calcined at 750 °C in an air environment to convert MOF precursors into CaO-based sorbents denoted as Ca-MOF-xh-y-O.

### 2.2. Characterization

X-ray diffraction data of the samples were collected by recording 2θ values ranging from 5° to 70°. Fourier-transform infrared (FT-IR) spectra were collected using a PerkinElmer Spectrum 100 FTIR spectrometer (PerkinElmer, MA, USA) and the self-supporting potassium bromide pallets in the wave number range from 450 to 4000 cm^−1^. **A** Field Emission Scanning Electron Microscope (FE-SEM, JEOL JSM-6700F, JEOL, Tokyo, Japan) was used to observe particle size and morphology where the sample was pretreated by a surface coating with Au to improve its electrical conductivity under the operation conditions of a 15 kV accelerating voltage and a 10 μA current. Thermal gravimetric analysis (TGA) measurements were performed on a TA Instrument Q500 (TA Instrument, New Castle, DE, USA) to analyze thermal properties from 25 °C to 800 °C at a rate of 5 °C min^−1^ under a continuous nitrogen gas flow. Nitrogen adsorption-desorption isotherms were collected on a ASAP 2020 (Micromeritics, GA, USA) at 77 K.

### 2.3. CO_2_ Adsorption Analysis

The multiple CO_2_ carbonation–calcinations of the synthesized Ca-MOF CO_2_ sorbents were measured using a TGA. Prior to the multiple cycles test, the samples were first tested by heating at a rate of 30 °C min^−1^ to 750 °C for activation. Next, the samples were tested at 450 °C for capturing CO_2_ in pure CO_2_ gas (99.99%) for 100 min and then heated to 550 °C for calcination in pure N_2_ gas (99.99%) for 150 min. In order to determine the stability, the carbonation–calcination process was repeated 10 times. In this research, the CO_2_ capture capacity of the sorbents was quantified using TGA according to the following equation:

Capacity = (W−W_0_)/W_0_ × 100% (1)

where W is the weight of the sorbent after CO_2_ adsorption and W_0_ is the initial weight of the sorbent.

## 3. Results and Discussion

### 3.1. Characterizations and Properties of Limestone and Ca-MOF 

**Figure 1:**
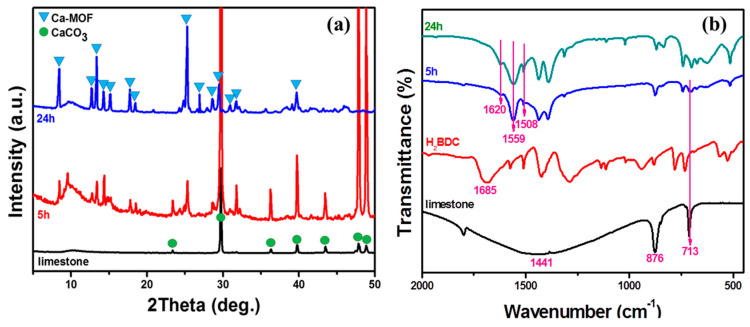
(**a**) XRD patterns and (**b**) FTIR spectra of limestone acidified in acetic acid at different acidification times with 10 M acetic acid to form Ca-based metal–organic framework (Ca-MOF)-5h-10 and Ca-MOF-24h-10.

Natural limestone is mainly composed of calcium carbonate. The morphology and composition of natural limestone are illustrated in Figure S1 (Supplementary Materials). Figure 1a shows the XRD patterns of rod Ca-MOF-xh-10 synthesized from natural limestone by a hydrothermal method via the assistance of acetic acid and the ligand solution of terephthalic acid (H_2_BDC) at different acidification times (x) with 10 M of acetic acid. The characteristic peaks of CaCO_3_ in limestone were indexed at 2θ= 23.2°, 29.8°, 36.3°, 39.7°, 43.3°, 47.8° and 48.9°. With increasing acidification time, Ca^2+^ was gradually released from the limestone and reacted with ligands to form Ca-MOF. When the acidification time increased up to 5 h, two distinct phases of CaCO_3_ and Ca-MOF were detected. Upon further increasing the acidification time to 24 h, it was found that limestone had been completely converted to Ca-MOF. The characteristic peaks of Ca-MOF around 2θ at 8.2°, 12.5°, 13.1°, 14.2°, 15.1°, 17.6°, 25.2° and 39.6° in Figure 1a further confirms this and corresponded to the data base of JCPDF NO.46-1873.

To identify the limestone and Ca-MOF-xh-10 structure, FTIR was used to analyze the adsorption peaks of the functional group (Figure 1b). The adsorption peaks of the limestone at 713 cm^−1^, 875cm^−1^ and the band between 1563 cm^−1^ and 1355 cm^−1^ were indexed as the in-plane bending of carbonate ion, out-of-plane bending vibration of carbonate ion and asymmetric stretching of carbonate ions, respectively [23]. The adsorption peak at 1685 cm^−1^ represented the characteristic adsorption of C=O bonds of COOH in H_2_BDC. As the acidification time increased, Ca^2+^ was gradually released from the limestone and bonded with deprotonated H_2_BDC to form Ca-MOF. However, the C=O peak at 1685 cm^-1^ disappeared in Ca-MOF-xh-10, which indicates that H^+^ was released from COOH in H_2_BDC. Strong C–O adsorption peaks at 1559 and 1620 cm^−1^ were observed, which can be assigned to the asymmetrical stretching vibration and the symmetrical stretching modes of C-O bonds in coordinated COO- groups, respectively [24]. The peak at 1508 cm^−1^ was assigned to the vibration of a benzene ring. The above results demonstrate the bonding between COO- of H_2_BDC and Ca^2+^. In contrast, Ca-MOF-5h-10 simultaneously showed two kinds of adsorption peaks at 1620 and 1559 cm^−1^ from coordinated COO groups in the Ca-MOF structure and 713 cm^−1^ from the carbonate ions of limestone, indicating that partial limestone was transformed into the Ca-MOF structure. However, a long acidification time can promote transformation, so the adsorption peak of Ca-MOF-24h-10 located at 713 cm^−1^ disappeared and the limestone was fully converted to Ca-MOF.

### 3.2. Formation Mechanism and Crystal Growth of Rod Ca-MOF

**Figure 2:**
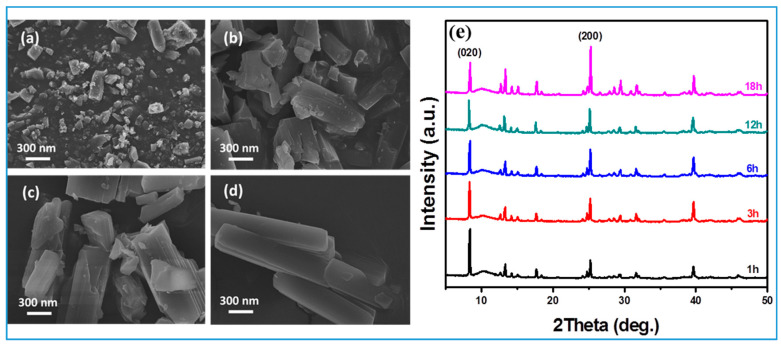
SEM images of Ca-MOF 24h-10 with different reaction times: (**a**) 10 min; (**b**) 1 h; (**c**) 6 h; (**d**) 18 h; (**e**) the corresponding XRD.

In order to understand the crystal growth of the rod Ca-MOF, acidic limestone was subjected to different reaction times from 10 min to 18 h. The SEM morphologies in Figure 2a show that 20–100 nm nanoparticles appeared in the initial reaction time of about 10 min. When the reaction time was 1 h, there were numerous nanoparticles along with irregular larger particles (Figure 2b). After 6 h, the embryonic form of the rod shape became visible together with the 100–200 nm particles on the surface in Figure 2c. The crystal continued to grow with the reaction time along the (200) direction to form the sheet-stack rod-like structure shown in Figure 2d, which coincided with the XRD patterns shown in Figure 2e, indicating that the intensity of (200) face became stronger as the reaction time increased from 1 to 18 h. The preferred crystal growth behavior was related to the different growth rates of the faces based on the different surface energies of all the crystal faces. In other words, the face with the highest surface energy exhibited the fastest growth rate. In our case, the (020) face possessed the highest surface energy among all the faces, and as a consequence, the crystal grew rapidly toward the direction of (020) to form a sheet morphology in the initial stage of Ca-MOF. Finally, the Ca-MOF formed a rod-like shape with a (200) face-oriented attachment growth.

**Scheme 2:**
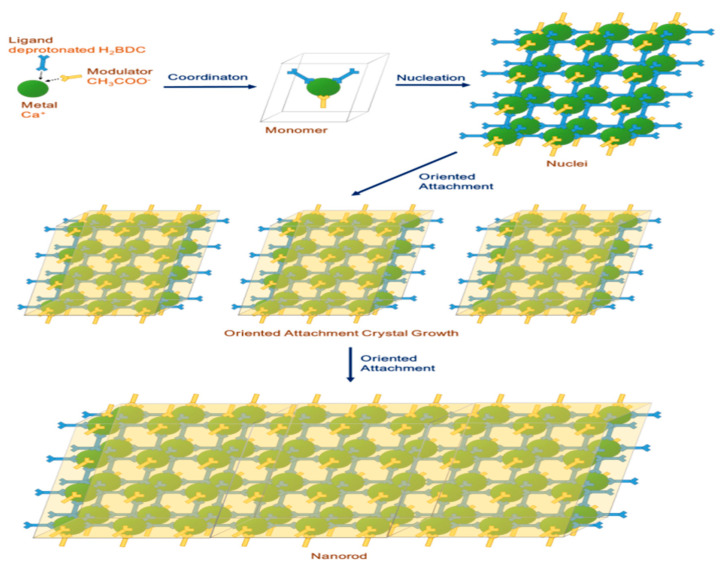
Mechanism for formation of rod Ca-MOF.

Based on the above results, we proposed a probable formation mechanism for rod Ca-MOF using oriented attachment growth, [25,26,27] as illustrated in Scheme 2. In the initial reaction process, a Ca^2+^ metal cation was first released from limestone (CaCO_3_) in the deprotonated CH_3_COOH solution and subsequently reacted with the deprotonated organic ligand terephthalic acid (H_2_BDC) generated in the polar solvent N, N-dimethylacetamide (DMA) to form Ca(BDC)(H_2_O)_3_, (i.e., Ca-MOF monomers). Later, the monomers nucleated to form Ca-MOF nuclei with a monoclinic crystal system in accordance with JCPDF NO.46-1873. The embryonic form of the rod shape can be observed after 6 h, indicating that coordination mode in the (200) direction is the preferable interaction for oriented attachment crystal growth over other directions. This can be explained by the fact that CH_3_COO^−^, by adding capping agents (modulators), has the same chemical functional group (COO–) as deprotonated H_2_BDC, so that the CH_3_COO^–^ modulators interfere with the coordination between the Ca^2+^ and deprotonated ligand H_2_BDC on the other faces, except in the (200) direction. Therefore, promoting the links between the Ca^2+^ metal and ligand H_2_BDC only occurred at the (200) and (−200) surfaces. The presence of CH3COO^−^ modulators with the COO- functional group is the key to enhancing the relatively fast rate of crystal growth in the (200) direction. Finally, rod-like Ca-MOF was obtained.

### 3.3. Characterizations of Rod Ca-MOF Structures Synthesized with Different Acetic Acid Concentrations

**Figure 3:**
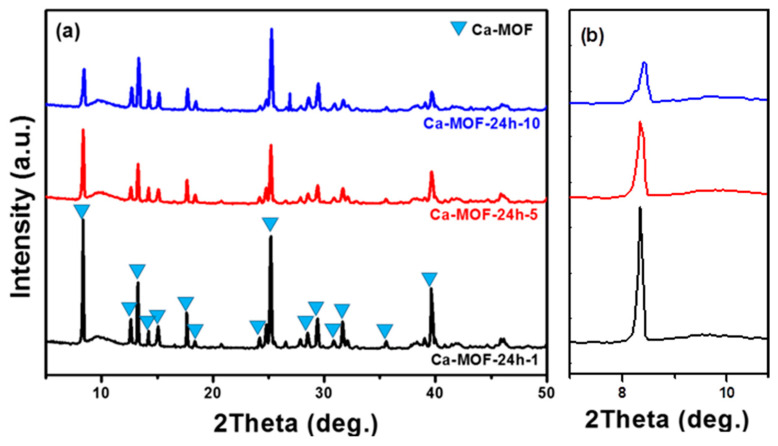
(**a**) XRD patterns of Ca-MOF-24h-y at different CH_3_COOH concentrations and (**b**) enlargement of the XRD pattern in selected 2 theta regions.

The above formation mechanism shows us that the rod-like Ca-MOF sorbent is strongly dependent on the CH_3_COO^-^ modulators, so the synthesis of rod-like Ca-MOF was further investigated using natural limestone under a hydrothermal process at different concentrations of acetic acid (1, 5 and 10 M), denoted as Ca-MOF-24h-y (y = 1, 5 and 10), respectively. The X-ray diffraction (XRD) in Figure 3a shows that as the CH_3_COOH concentration decreased from 10 M to 1 M, the peaks of Ca-MOF-24h-1 became much sharper and more symmetrical than those of Ca-MOF-24h-10 shown in Figure 3b. This phenomenon illustrated that low-concentration CH_3_COOH promoted better crystallinity and larger crystal sizes of Ca-MOF, which is consistent with the above result of the CH_3_COO^−^ modulators as capping agents to interfere with the coordination between the Ca^2+^ and deprotonated ligand H_2_BDC, inhibiting the growth of Ca-MOF.

**Figure 4:**
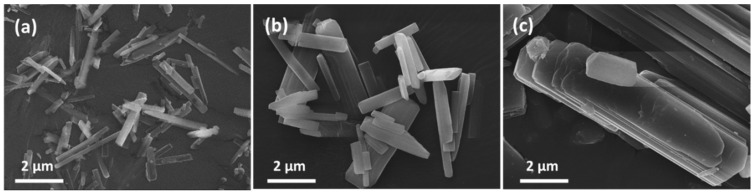
SEM images of different CH_3_COOH concentrations. (**a**) Ca-MOF-24h-10 (**b**) Ca-MOF-24h-5 and (**c**) Ca-MOF-24h-1.

Figure 4 shows the SEM morphology of Ca-MOF-24h-y synthesized at different CH_3_COOH concentrations. All Ca-MOF-24h-y samples exhibited a uniform crystal size with a smooth surface. Ca-MOF-24h-10 showed a needle-like shape with a length of 0.5–2 μm and width of 100–300 nm. As the concentration of CH_3_COOH decreased, the length and the width of the Ca-MOF crystals gradually grew to a larger size. The sheet-like crystals were also stacked to form rod-like Ca-MOF-24h-1 crystals with a length of 5–10 μm and width of 1–2 μm. These results illustrated that the morphology of Ca-MOF was strongly correlated with the concentration of CH_3_COOH molecules. In the high concentration of the CH_3_COOH reaction system, as a competitor to deprotonated ligand H_2_BDC, more CH_3_COO^-^ may tend to react with Ca^2+^, which causes the inhibition of the coordinating reaction of H_2_BDC with the metal Ca^2+^ ion. Therefore, the formation and crystal growth of the Ca-MOF structure would be affected, resulting in a Ca-MOF with a smaller size and lower crystallite due to the inhibition of the crystal growth by the coordination of Ca^2+^ and CH_3_COO^−^. In contrast, in a low-CH_3_COOH concentration system, the Ca-MOF crystal would gradually grow to a larger, more well-oriented crystal after a longer time.

### 3.4. Pore Formation of Rod Ca-MOF Network

As the Ca-MOF was used as an intermediate-temperature CO_2_ sorbent, it was subjected to thermal treatment. In order to understand the effect of calcination temperature on the formation of pores, the thermal property of Ca-MOF-24 h-1 was analyzed under continuous N_2_ gas flow with a heating rate of 5 °C min^−1^ from room temperature to 720 °C. The result in Figure S2 reveals that there were three stages of weight loss. In the first stage, a weight percent change around 20 wt% at 75 ~ 130 °C was attributed to the loss of water molecules on the surface. After that, the phase transfer from Ca-MOF to Ca-MOF* occurred at 150 °C, as indicated in the XRD of Figure S3 [28]. The Ca-MOF* phase presented a relatively stable structure with only slight weight loss up to 400 °C. The FT-IR spectra in Figure S4 also demonstrate that the characteristic peaks of ligand H_2_BDC in Ca-MOF fully disappeared, while the peaks at 713 cm^−1^, 875 cm^−1^ and the band between 1563 cm^−1^–1355 cm^−1^ were also detected because of the presence of the in-plane bending of the carbonate ions, out-of-plane bending vibration of the carbonate ions and the asymmetric stretching of carbonate ions, respectively [19]. However, as the temperature continued to increase higher than 550 °C, severe weight loss occurred since the framework gradually decomposed, indicating that Ca-MOF was completely decomposed and transferred to CaCO_3_. Finally, the last part of the weight loss occurred between 500–590 °C. The phase gradually transferred from CaCO_3_ to CaO, as shown in Figure S3.

**Figure 5:**
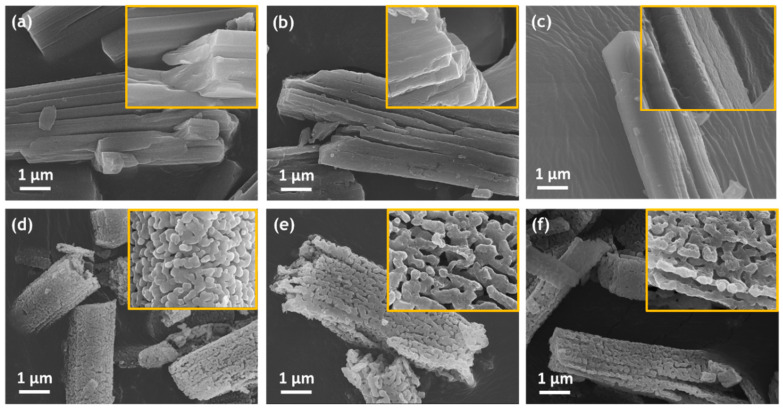
SEM images of Ca-MOF-24h-1 as-synthesized and calcined at different temperatures. (**a**) As-synthesized: (**b**) C150; (**c**) C300; (**d**) C500; (**e**) C600; (**f**) C750.

Figure 5 shows the SEM microstructure evolution of Ca-MOF as the heating temperature increases. The rod-like Ca-MOF-24 h-1 was still a complete solid with a smooth surface below 400 °C, which is shown in Figure S2, where no obvious weight loss was detected. However, as the Ca-MOF was heated over 400 °C, i.e., at 500 °C, a rod-like porous-network Ca-MOF-24h-1 was observed due to the disappearance of the ligands, where the network Ca-MOF was composed of 200–400 nm irregular nanoparticles. With increasing temperature, each nanoparticle gathered together because of sintering and gradually formed a hollow network with 20–50 nm pores inside a rod-like porous network. The pore size was further enlarged at 600 °C due to the decomposition of CaCO_3_ into CaO, as evidenced by the XRD in Figure S2. The higher temperature above 600 °C shows no obvious change in Figure 5f.

**Figure 6:**
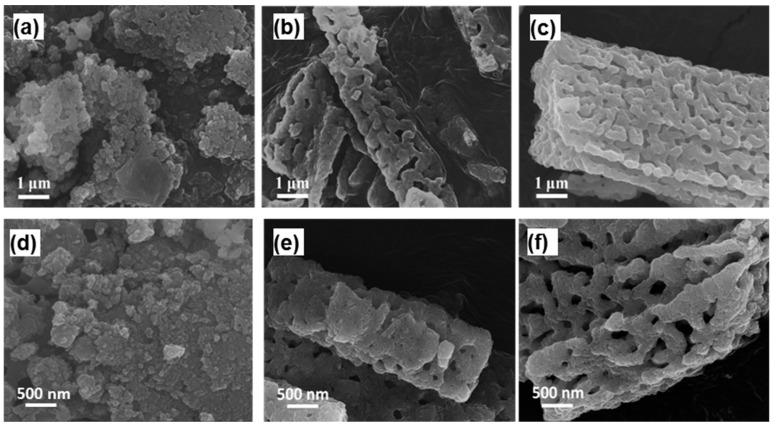
SEM images of as-calcined samples for (**a**) Ca-MOF-24h-10-O, (**b**) Ca-MOF-24h-5-O and (**c**) Ca-MOF-24h-1-O. After the 10^th^ carbonation–calcination cycle, the SEM morphology images of (**d**) Ca-MOF-24h-10-O, (**e**) Ca-MOF-24h-5-O and (**f**) Ca-MOF-24h-1-O.

When the as-synthesized powders were calcined at 750 °C for 2 h (denoted as Ca-MOF-24h-10-O, Ca-MOF-24h-5-O and Ca-MOF-24h-1-O, where “O” indicates the calcination), the Ca-MOF completely decomposed into CaO during the calcination process. The SEM images of calcined powders in Figure 6a show that the Ca-MOF-24h-10-O synthesized at a high CH_3_COOH concentration was unstable after calcination and entirely collapsed into uniform nanoparticles, with irregular shapes of approximately 100–300 nm in diameter. The overall structure displayed a powder compact structure, resulting in particle aggregation. In contrast, the Ca-MOF-24h-1-O synthesized at a lower CH_3_COOH concentration still kept an integral rod-like shape with 20–50 nm pores (Figure 6c), which can be explained by the better crystallinity of the as-synthesized Ca-MOF-24h-1 powders to offer a rigid framework for maintaining a rod-like crystal during the calcination process.

**Figure 7:**
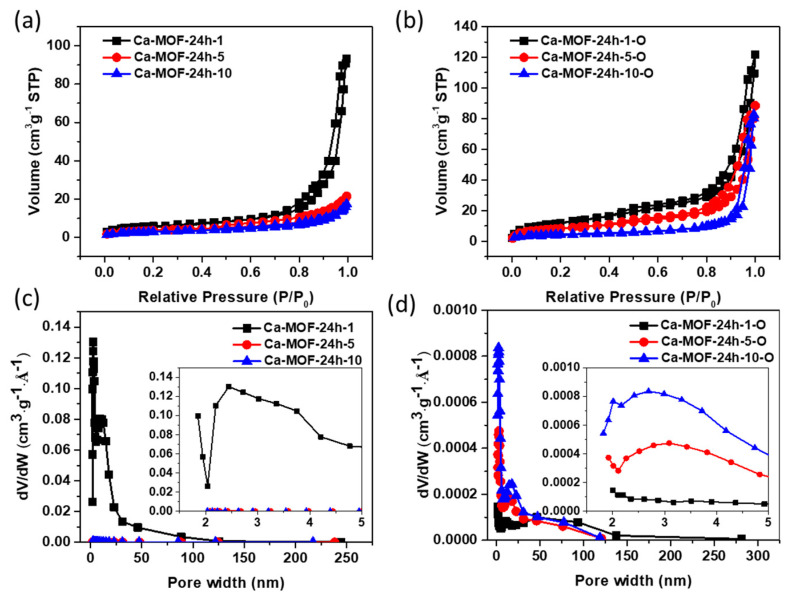
N_2_ adsorption–desorption isotherms of (**a**) Ca-MOF-24h-y and (**b**) Ca-MOF-24h-y-O; mesopore size distribution curve of (**c**) Ca-MOF-24h-y and (**d**) Ca-MOF-24h-y-O.

The specific surface areas of Ca-MOF-24h-y and Ca-MOF-24h-y-O were investigated by N_2_ adsorption–desorption isotherms. As shown in Figure 7a,b, both Ca-MOF-24h-y and Ca-MOF-24h-y-O showed typical type-III isotherms. Figure 7c,d show the pore distribution of Ca-MOF-24h-y and Ca-MOF-24h-y-O, respectively. The pore size distribution of Ca-MOF-24h-y is almost at ca. 2–50 nm, which is defined as mesoporous. In contrast, after heat treatment, the pore size distribution of Ca-MOF-24h-y-O gradually increased above 50 nm. The analysis of surface area for Ca-MOF-24h-y and Ca-MOF-24h-y-O was further demonstrated in Table 1, which showed that there are more mesopores in Ca-MOF-24 h-y, but the meso/macro pores are dominant in the calcined Ca-MOF-24h-y-O sample.

**Figure 8:**
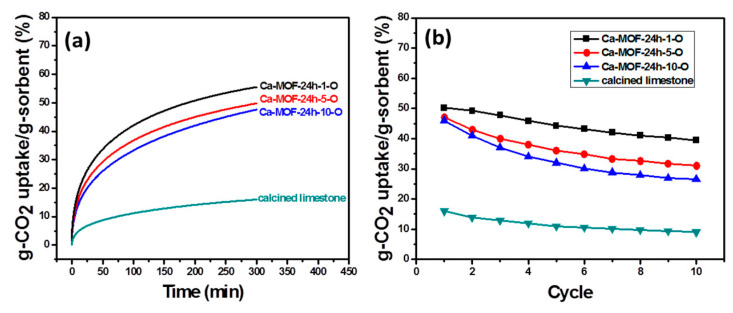
(**a**) CO_2_ capture kinetics at 450 °C and (**b**) multiple carbonation–calcination cycles of Ca-MOF-24h-y-O and calcined limestone.

To test the CO_2_ capture capacity, Ca-MOF-24 h-y-O was used for the carbonation adsorption under intermediate-temperature conditions (450 °C) (Figure 8a). The results showed that the Ca-MOF-24h-1-O sorbent displayed the highest CO_2_ capture capacity with about 56 wt% in 6 h. In addition, the carbonation kinetics and capture amount of all the Ca-MOF-24h-y-O sorbents far exceeded those of calcined limestone particles. The positive effect on the CO_2_ capture capacity and carbonation kinetics of Ca-MOF-24h-y-O sorbents can be ascribed to the porous framework of the rod-like crystal, which allowed for the rapid diffusion of CO_2_ into the interior of the sorbent. Figure 8b shows the multiple carbonation–calcination cycles of three Ca-MOF-24h-y-O sorbents and calcined limestone, where the carbonation was performed at 450 °C in pure CO_2_ for carbonation and calcination at 550 °C in 100% N_2_. The CO_2_ capture capacity of the Ca-MOF-24h-1-O sorbent decreased from 52% to 41% after 10 cycles, which showed an excellent stability of 80% that was higher than that of the other two calcined limestone sorbents. In contrast, a lower capture amount and poor cyclic performance was observed for the Ca-MOF-24 h-10-O sorbent, which can be attributed to particle aggregation after the porous framework was destroyed due to calcination. This adsorption behavior is intensively related to the formation of a porous framework because the pores can reduce the aggregation from the partial sintering between CaO/CaCO_3_ particles. Our results demonstrate that limestone can be treated with acetic acid to transform it into a porous rod Ca-MOF as CO_2_ adsorbent to improve its CO_2_ capture performance. However, as it was applied in an industrial real calcium looping process for CO_2_ capture at intermediate temperature or high temperatures, rod CO_2_ adsorbents with porous networks and connected multi-channels for enhancing gas/solid contact and mass/heat transfer can overcome interparticle forces during the looping process. In addition, serious aggregation can be avoided by increasing the particle size from the present 5–10 um to 50 um or larger via modulating the reaction time and acetic acid concentration.

To confirm the reason for the high CO_2_ capture capacity and stability of Ca-MOF-24h-1-O, SEM morphology images of Ca-MOF-24h-y-O after multiple carbonation–calcination cycles are illustrated in Figure 6d–f. The calcined limestone presented a larger bulk size, being approximately 1–3 μm in diameter. At 24 h, Ca-MOF-24 h-10-O exhibited the smallest particles, being approximately 50–100 nm in diameter with a round shape. These small particles stacked with each other randomly to form larger particles of about 0.5–1 μm. The numbers of active sites for CO_2_ adsorption were reduced and the dense surface made CO_2_ diffusion into the particle difficult. On the other hand, the Ca-MOF-24h-1-O sorbent still preserved a porous network structure after 10 cycles, which allowed for CO_2_ diffusion through entire particles from the surface to enhance the carbonation kinetics. Consequently, the Ca-MOF-24h-1-O sorbent exhibited a better CO_2_ capture efficiency over 10 cycles.

## 4. Conclusions

In summary, the transfer of rod Ca-MOF from limestone with the assistance of acetic acid was successfully synthesized by a hydrothermal method. The crystal size of rod Ca-MOF can be controlled by the concentration of CH_3_COOH. After calcination, the Ca-MOF-O exhibits a porous network structure, which can be used as an excellent sorbent in CO_2_ capture at an intermediate temperature. The results indicate that the Ca-MOF-24h-1-O transferred from a larger crystal displays a pore distribution of 50–100nm in the porous network with 5–20 nm pores, which allows for the rapid diffusion of CO_2_ into the interior of the sorbent and enhances the CO_2_ capture capacity of about 52 wt% and the high CO_2_ capture stability of approximately 80% during 10 carbonation–calcination cycles. The porous network structure of rod CaO transferred from Ca-MOF, synthesized to promote CO_2_ capture performance, has potential in environmental applications.

## Figures and Tables

**Table 1:** The specific surface area and pore size distribution of Ca-MOF-24 h-y and Ca-MOF-24 h-y-O.

Sample	Ca-MOF-24h-1	Ca-MOF-24h-5	Ca-MOF-24h-10	Ca-MOF-24h-1-O	Ca-MOF-24h-5-O	Ca-MOF-24h-10-O
S_BET_ (m^2^g^−1^)	21	13	11	48	30	15
S_micro_/S_BET_ (%)	14	3	5	7	5	14
S_meso/macro_/S_BET_ (%)	86	97	95	93	95	86

S_BET_: the BET surface area; S_micro_: micropore of surface area; S_meso/macro_: meso/macropore of surface area.

## Supplementary Materials

The following are available online at https://www.mdpi.com/1996-1944/13/19/4297/s1, Figure S1: Morphology and characterization of limestone powder; Figure S2: TGA curves of thermal property about Ca-MOF-24h-1; Figure S3: XRD patterns about Ca-MOF-24h-1 of as-synthesized and calcined at different temperatures; Figure S4: FT-IR spectra about Ca-MOF-24h-1 of as-synthesized and calcined at different temperatures.
