# Supplementary material for: Synthesis and Formation Mechanism of Limestone-Derived Porous Rod Hierarchical Ca-based Metal–Organic Framework for Efficient CO2 Capture
Source: Materials (Basel). 2020 Sep 26;13(19):4297. doi: 10.3390/ma13194297 (PMC7579375; doi:10.3390/ma13194297)
Supplement: Supplementary file 1 [file materials-13-04297-s001.pdf]

materials

MDPI

Supplementary Materials: Synthesis and Formation Mechanism of Limestone-Derived Porous Rod Hierarchical Ca-based Metal–Organic Framework for Efficient CO_2 Capture

Po-Hsueh Chang [1,†], Hua-Pei Hsu [2], Szu-Chen Wu [2,†] and Cheng-Hsiung Peng [1,*]

1. Department of Chemical and Materials Engineering, Minghsin University of Science and Technology, Xinxing Road, Xinfeng, Hsinchu 30401, Taiwan; pohsueh.chang@gmail.com
2. Department of Materials Science and Engineering, National Chiao Tung University, 1001 University Road, Hsinchu 30010, Taiwan; ivy810823@gmail.com (H.-P.H.); s320431@hotmail.com (S.-C.W.)

* Correspondence: chpeng@must.edu.tw; Tel.: +886-3-5593-142 (ext. 2119) or +886-933-961471; Fax: +886-3-5593-377

† Equal contribution.

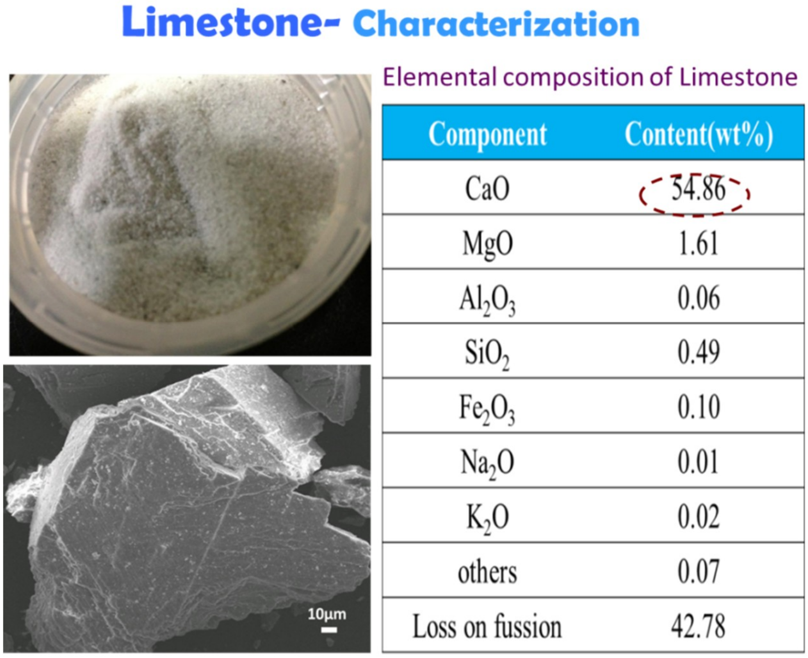

Component	Content(wt%)
CaO	54.86
MgO	1.61
Al_2O_3	0.06
SiO_2	0.49
Fe_2O_3	0.10
Na_2O	0.01
K_2O	0.02
others	0.07
Loss on fussion	42.78

Figure S1. Morphology and characterization of limestone powder

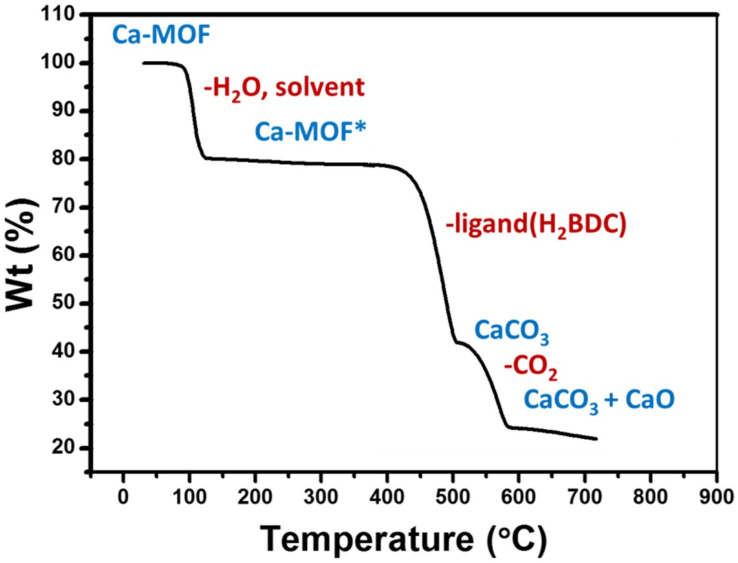

Figure S2. TGA curves of thermal property about Ca-MOF-24h-1.

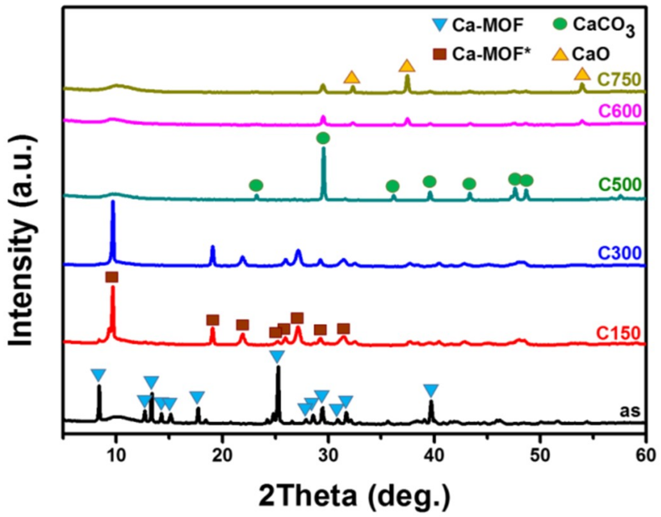

Figure S3. XRD patterns about Ca-MOF-24h-1 of as-synthesized and calcined at different temperatures.

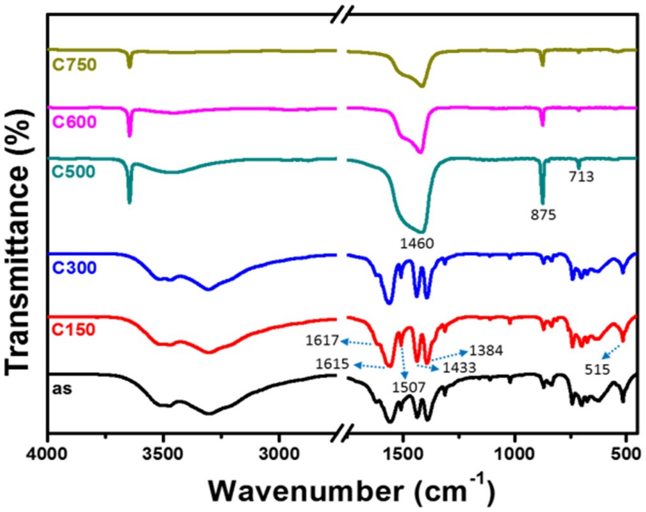

Figure S4. FT-IR spectra about Ca-MOF-24h-1 of as-synthesized and calcined at different temperatures.
